# Supplementary material for: Viral dynamics of acute SARS-CoV-2 infection and applications to diagnostic and public health strategies
Source: PLoS Biol. 2021 Jul 12;19(7):e3001333. doi: 10.1371/journal.pbio.3001333 (PMC8297933; doi:10.1371/journal.pbio.3001333)
Supplement: S1 Fig — Histogram of the proportion of consecutive tests that are within n days of one another up to n = 12 days. Only 12 of 2,343 intervals (0.05%) exceeded 12 days. Underlying data are available at https://github.com/gradlab/CtTrajectories/tree/main/figure_data/FigS1. (PDF) [file pbio.3001333.s001.pdf]

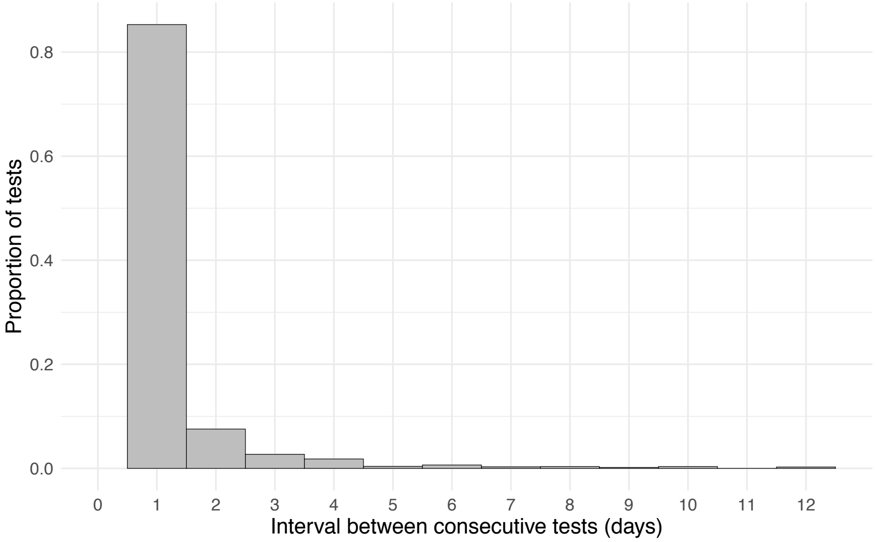

S1 Fig. Distribution of intervals between consecutive tests. Histogram of the proportion of consecutive tests that are within *n* days of one another up to *n* = 12 days. Only 12 of 2343 intervals (0.05%) exceeded 12 days. Underlying data are available at https://github.com/gradlab/CtTrajectories/tree/main /figure_data/FigS1[10]
