# Supplementary material for: Viral dynamics of acute SARS-CoV-2 infection and applications to diagnostic and public health strategies
Source: PLoS Biol. 2021 Jul 12;19(7):e3001333. doi: 10.1371/journal.pbio.3001333 (PMC8297933; doi:10.1371/journal.pbio.3001333)
Supplement: S5 Fig — Points depict observed Ct values, which are connected with lines to better visualize patterns. Individuals with presumed acute infections are in red. All others are in black. Underlying data are available at https://github.com/gradlab/CtTrajectories/tree/main/data. (PDF) [file pbio.3001333.s005.pdf]

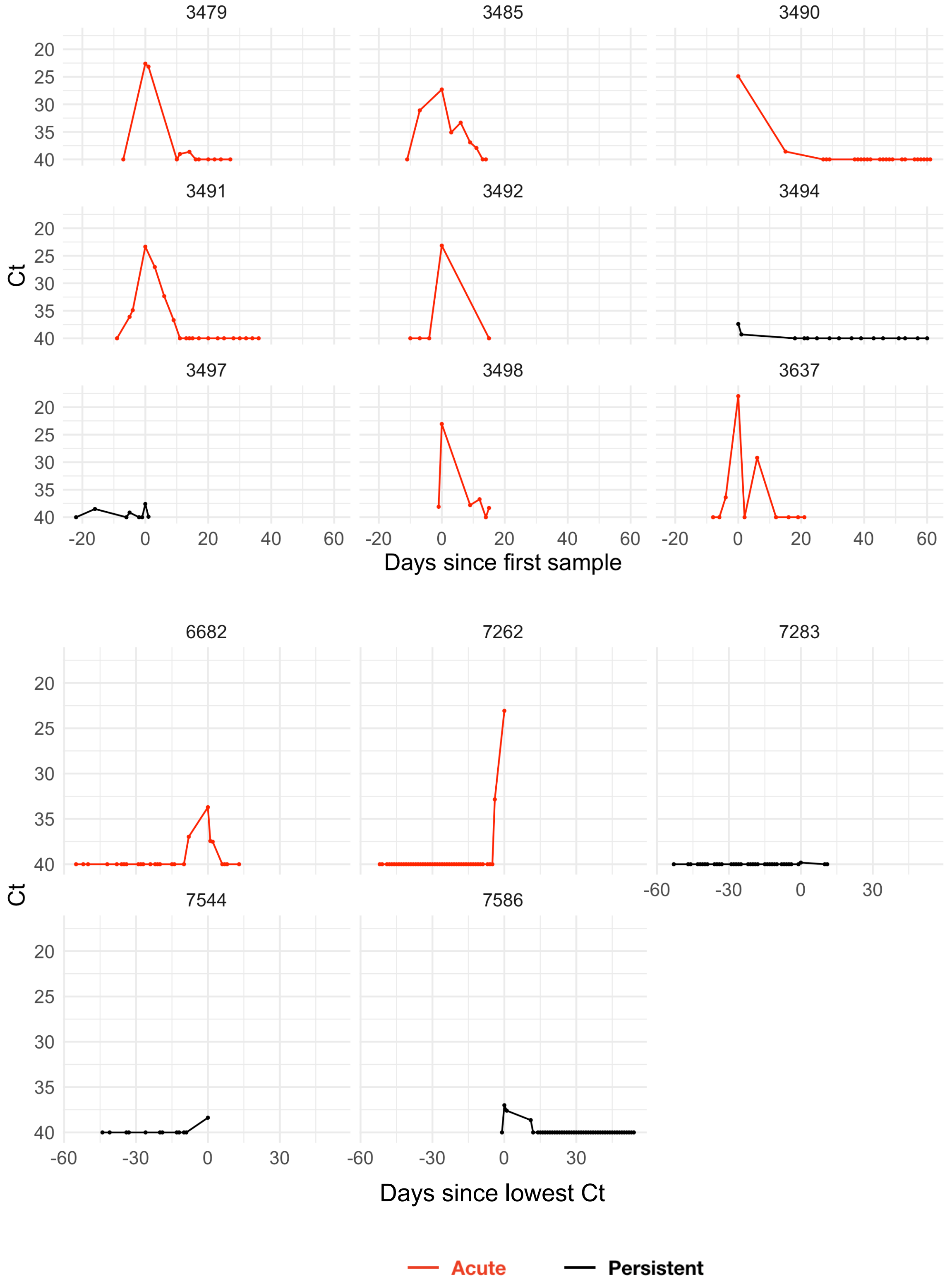

S5 Fig. Observed Ct values from the study participants (4/4). Points depict observed Ct values, which are connected with lines to better visualize trends. Individuals with presumed acute infections are marked in red. All others are in black. Underlying data are available at https://github.com/gradlab/CtTrajectories/tree/main /data[10]
