# Supplementary material for: Viral dynamics of acute SARS-CoV-2 infection and applications to diagnostic and public health strategies
Source: PLoS Biol. 2021 Jul 12;19(7):e3001333. doi: 10.1371/journal.pbio.3001333 (PMC8297933; doi:10.1371/journal.pbio.3001333)
Supplement: S6 Fig — E[Ct] is the expected Ct value on a given day. The Ct begins at the limit of detection, then declines from the time of infection (to) to the peak at χ cycles below the limit of detection at time tp. The Ct then rises again to the limit of detection after tr days. The model incorporating these parameter values used to generate this piecewise curve is given in the equation for E[Ct(t)] in S1 Text (Supplemental Methods, under the heading “Model fitting”). (PDF) [file pbio.3001333.s006.pdf]

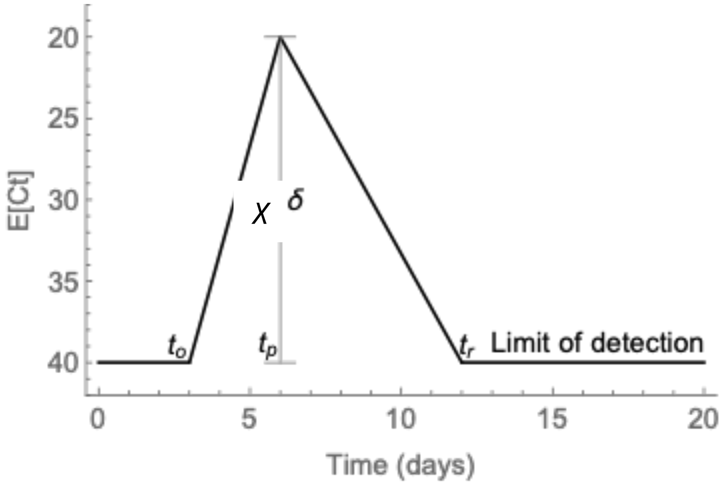

S6 Fig. A theoretical Ct trajectory. E[Ct] is the expected Ct value on a given day. The Ct begins at the limit of detection, then declines from the time of infection (t_o) to the peak at χ cycles below the limit of detection at time t_p. The Ct then rises again to the limit of detection after t_r days. The model incorporating these parameter values used to generate this piecewise curve is given in Equation S1 (**Methods**).
