# Supplementary material for: Viral dynamics of acute SARS-CoV-2 infection and applications to diagnostic and public health strategies
Source: PLoS Biol. 2021 Jul 12;19(7):e3001333. doi: 10.1371/journal.pbio.3001333 (PMC8297933; doi:10.1371/journal.pbio.3001333)
Supplement: S7 Fig — (A) To calculate the effective sensitivity of a test intended to screen infectious individuals before a gathering, we first drew 1,000 viral trajectories as defined by the peak Ct, proliferation time, and clearance time from the fitted model (step 1, with 3 draws illustrated in red, green, and blue). We restricted to only individuals with viral concentrations above the infectiousness threshold (here the threshold is at Ct = 30, requiring us to omit the fourth entry). Then, we assigned detectability onset times—i.e., the times at which the trajectories could first be detected by PCR with limit of detection at 40 Ct—according to a standard uniform distribution, ensuring that the trajectories surpassed the infectiousness threshold at some point during the gathering (step 2). The onset times are depicted as colored dots. Finally, for a test administered some span of time prior to the event, we calculated the fraction of these infections the test would detect—this is the effective sensitivity (step 3). For a test administered at the time marked by the vertical black bar, the green trajectory would be detected by both PCR and a rapid test, the red trajectory would be detected by PCR but not a rapid test, and the blue trajectory would not be detected by either test. (B) To calculate the number of people who would arrive at a gathering while infectious, we performed a similar procedure. First, given a gathering size N and prevalence of PCR-detectable individuals p, we drew η trajectories from the fitted model where η ~ Binomial(N, p). Three such draws are depicted in step 1; note that, here, the only requirement was that the individuals were detectable (not necessarily infectious) at the time of the gathering, and so the previously omitted value could now be chosen. Then, as before, detectability onset times (colored dots) were drawn from a uniform distribution ensuring that the individuals were PCR-detectable at the time of the gathering (2). Finally, in step 3, the number [file pbio.3001333.s007.pdf]

A)

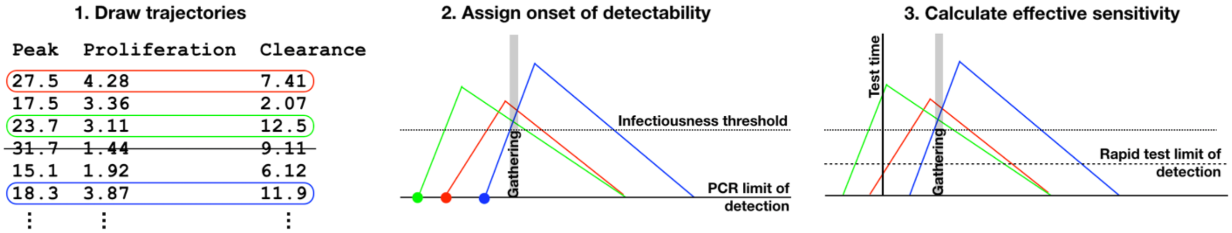

B)

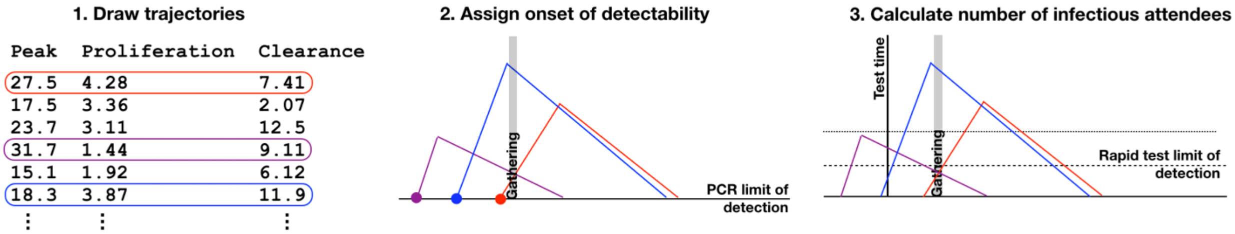

S7 Fig. Schematics illustrating calculations for effective sensitivity the expected number of infectious attendees at a gathering given a pre-gathering test. A) To calculate the effective sensitivity of a test intended to screen infectious individuals before a gathering, we first drew 1,000 viral trajectories as defined by the peak Ct, proliferation time, and clearance time from the fitted model (1, with three draws illustrated in red, green, and blue). We restricted to only individuals with viral concentrations above the infectiousness threshold (here the threshold is at Ct = 30, requiring us to omit the fourth entry). Then, we assigned detectability onset times – that is, the times at which the trajectories could first be detected by PCR with limit of detection at 40 Ct – according to a standard uniform distribution, ensuring that the trajectories surpass the infectiousness threshold at some point during the gathering (2). The onset times are depicted as colored dots. Finally, for a test administered some span of time prior to the event, we calculate the fraction of these individuals who the test would screen – this is the effective sensitivity (3). For a test administered at the time marked by the vertical black bar, the green trajectory would be screened by both PCR and a rapid test, the red trajectory would be screened by PCR but not a rapid test, and the blue trajectory would not be screened by either test. B) To calculate the number of people who would arrive at a gathering while infectious, we perform a similar procedure. First, given a gathering size *N* and prevalence of PCR-detectable individuals *p*, we draw η trajectories from the fitted model where $\eta \sim \text{Binomial}(N, p)$. Three such draws are depicted in pane (1); note that here, the only requirement is that the individuals are detectable (not necessarily infectious) at the time of the gathering, and so the previously omitted value can now be chosen. Then, as before, detectability onset times (colored dots) are drawn from a uniform distribution ensuring that the individuals are PCR-detectable at the time of the gathering (2). Finally, in pane (3), the number of infectious individuals who would attend the gathering in the absence of a pre-gathering test are counted (in this case just the blue trajectory) as well as the number of individuals who would attend the event given a pre-gathering test. Here, the blue trajectory would be screened by a PCR test but not a rapid test at the test time depicted by the vertical black bar. The purple trajectory would be detected by both a rapid test and a PCR test, yet it would not have been infectious at the gathering (in fact, its trajectory never surpasses the infectiousness threshold depicted here). The green trajectory would not be detected by either test but also would not have arrived at the gathering while infectious since it has a relatively late onset time. Repeating this procedure for many simulated gatherings gives an estimate of the expected number of infectious people who would arrive at a gathering given a pre-gathering testing protocol.
