# Supplementary material for: Viral dynamics of acute SARS-CoV-2 infection and applications to diagnostic and public health strategies
Source: PLoS Biol. 2021 Jul 12;19(7):e3001333. doi: 10.1371/journal.pbio.3001333 (PMC8297933; doi:10.1371/journal.pbio.3001333)
Supplement: S8 Fig — Posterior distributions obtained from 10,000 posterior draws from the distributions for peak Ct value (A), duration of the proliferation stage (infection detection to peak Ct) (B), duration of the clearance stage (peak Ct to resolution of acute RNA shedding) (C), and total duration of acute shedding (D) across the 46 individuals with a verified infection. The mean Ct trajectory corresponding to the mean values for peak Ct, proliferation duration, and clearance duration is depicted in (E) (solid lines), where shading depicts the 90% credible interval. Underlying data are available at https://github.com/gradlab/CtTrajectories/tree/main/output/params_df_combined.csv. (PDF) [file pbio.3001333.s008.pdf]

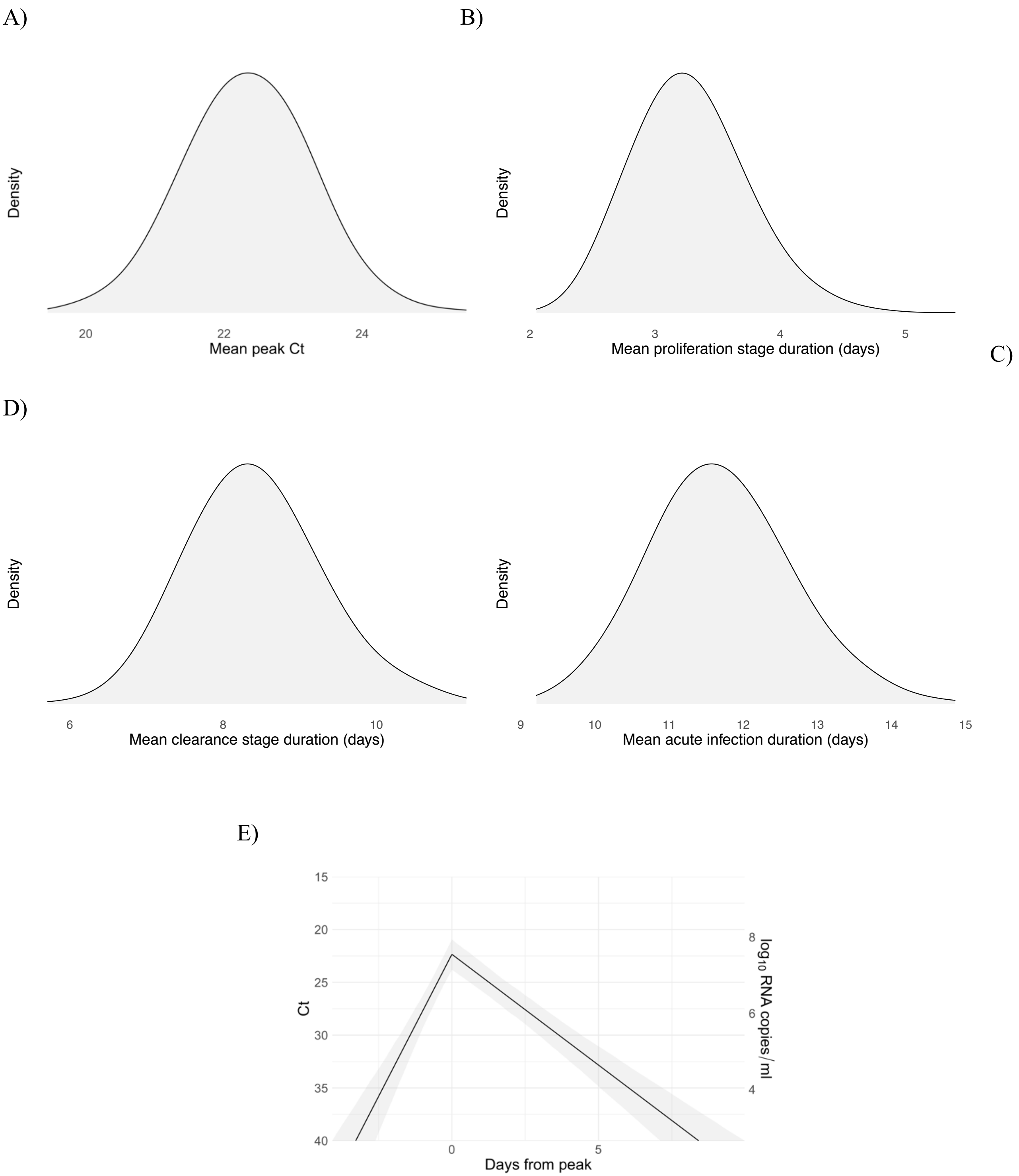

S8 Fig. Mean peak Ct value and waiting time distributions for individuals with acute infections. Posterior distributions obtained from 10,000 posterior draws from the distributions for peak Ct value (A), duration of the proliferation stage (infection detection to peak Ct, B), duration of the clearance stage (peak Ct to resolution of acute RNA shedding, C), and total duration of acute shedding (D) across the 46 individuals with a verified infection. The mean Ct trajectory corresponding to the mean values for peak Ct, proliferation duration, and clearance duration is depicted in (E) (solid lines), where shading depicts the 90% credible interval. Underlying data are available at https://github.com/gradlab/CtTrajectories/tree/main/output/params_df_combined.csv[10]
