# Supplementary material for: Viral dynamics of acute SARS-CoV-2 infection and applications to diagnostic and public health strategies
Source: PLoS Biol. 2021 Jul 12;19(7):e3001333. doi: 10.1371/journal.pbio.3001333 (PMC8297933; doi:10.1371/journal.pbio.3001333)
Supplement: S9 Fig — Underlying data are available at https://github.com/gradlab/CtTrajectories/tree/main/output/params_df_split.csv. (PDF) [file pbio.3001333.s009.pdf]

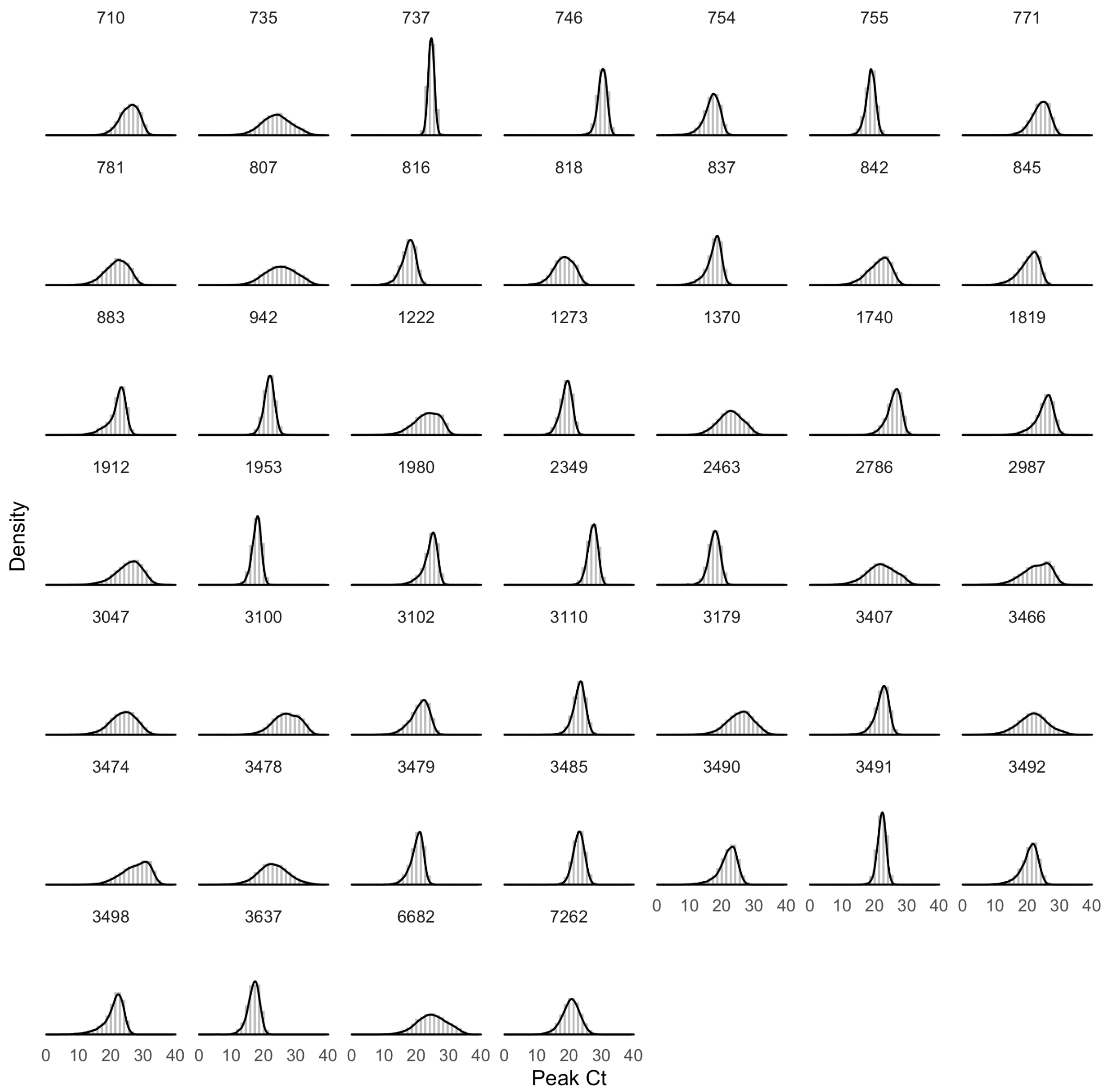

S9 Fig. Posterior peak Ct value distributions for the 46 individuals with acute infections. Underlying data are available at https://github.com/gradlab/CtTrajectories/tree/main/output/params_df_split.csv[10]
