# Supplementary material for: Viral dynamics of acute SARS-CoV-2 infection and applications to diagnostic and public health strategies
Source: PLoS Biol. 2021 Jul 12;19(7):e3001333. doi: 10.1371/journal.pbio.3001333 (PMC8297933; doi:10.1371/journal.pbio.3001333)
Supplement: S12 Fig — Thin grey lines depict 500 sampled trajectories. Points represent the observed data, with symptomatic individuals represented in red and asymptomatic individuals in blue. Underlying data are available at https://github.com/gradlab/CtTrajectories/tree/main/output/params_df_split.csv (lines) and https://github.com/gradlab/CtTrajectories/tree/main/data (points). (PDF) [file pbio.3001333.s012.pdf]

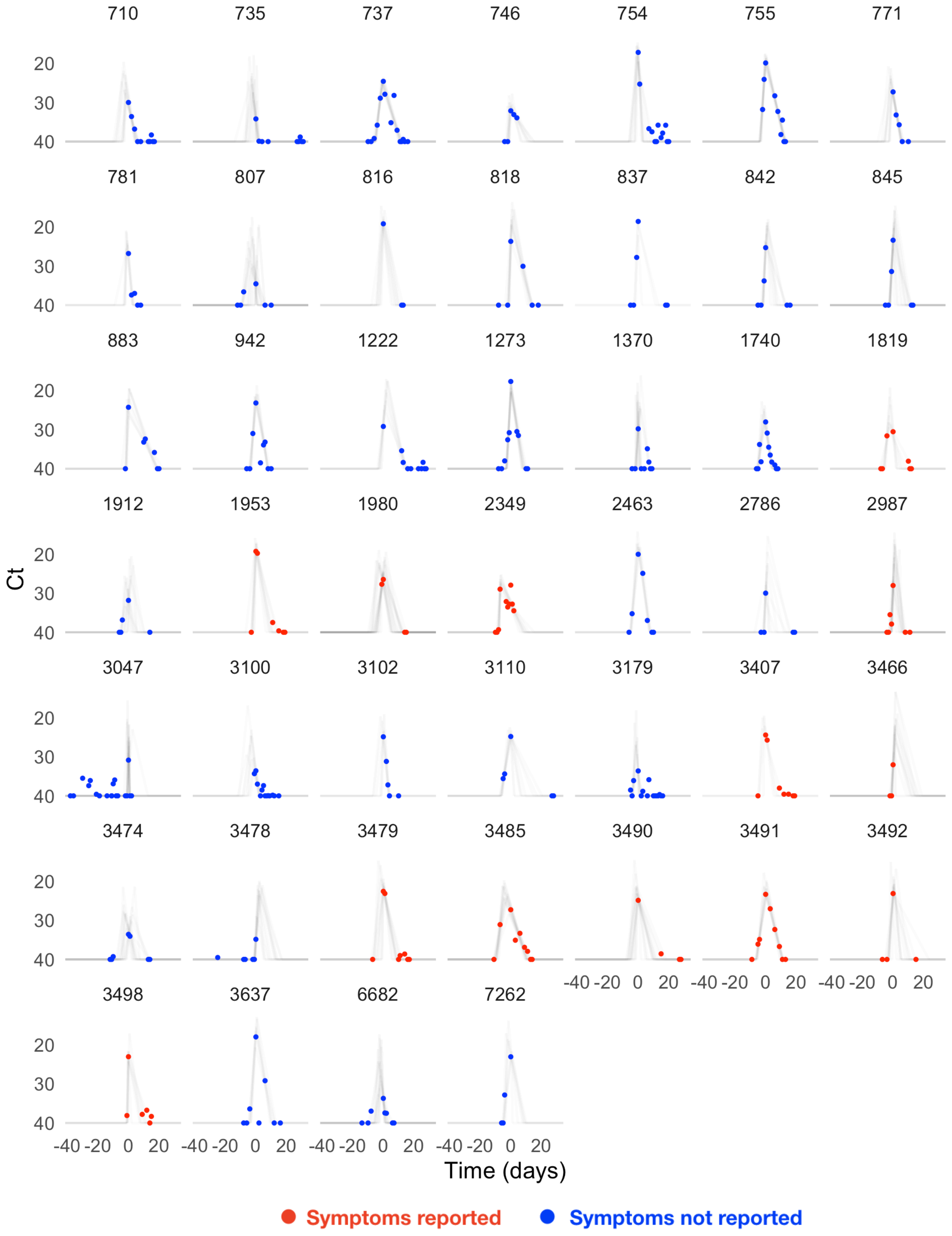

S12 Fig. Best-fit Ct trajectories for the 46 individuals with acute infections. Thin grey lines depict 500 sampled trajectories. Points represent the observed data, with symptomatic individuals represented in red and asymptomatic individuals in blue. Underlying data are available at Underlying data are available at https://github.com/gradlab/CtTrajectories/tree/main/output/params_df_split.csv[10] (lines) and https://github.com/gradlab/CtTrajectories/tree/main/data[10] (points)
