# Supplementary material for: Viral dynamics of acute SARS-CoV-2 infection and applications to diagnostic and public health strategies
Source: PLoS Biol. 2021 Jul 12;19(7):e3001333. doi: 10.1371/journal.pbio.3001333 (PMC8297933; doi:10.1371/journal.pbio.3001333)
Supplement: S13 Fig — Histograms (grey bars) of 10,000 posterior draws from the distributions for peak Ct value (A), time from onset to peak (B), time from peak to recovery (C), and total duration of infection (D) across the 46 individuals with an acute infection. Grey curves are kernel density estimators to more clearly exhibit the shape of the histogram. Black curves represent the best-fit normal (A) or gamma (B–D) distributions to the histograms. The duration of infection is the sum of the time from onset to peak and the time from peak to recovery. The best-fit normal distribution to the posterior peak Ct value distribution had mean 22.3 and standard deviation 4.2. The best-fit gamma distribution to the proliferation stage duration had shape parameter 2.3 and inverse scale parameter 0.7. The best-fit gamma distribution to the clearance stage duration had shape parameter 2.4 and inverse scale parameter 0.3. The best-fit gamma distribution to the total duration of infection had shape parameter 4.3 and inverse scale parameter 0.4. Alternatively, the proliferation, clearance, and total duration of infection distributions can be summarized as log-normal distributions. The best-fit log-normal distribution to the proliferation stage duration had location parameter μ = 0.93 and scale parameter σ = 0.82. The best-fit log-normal distribution to the clearance stage duration had location parameter μ = 1.9 and scale parameter σ = 0.83. The best-fit log-normal distribution to the total duration of infection had location parameter μ = 2.3 and scale parameter σ = 0.53. Underlying data are available at https://github.com/gradlab/CtTrajectories/tree/main/output/params_df_split.csv. (PDF) [file pbio.3001333.s013.pdf]

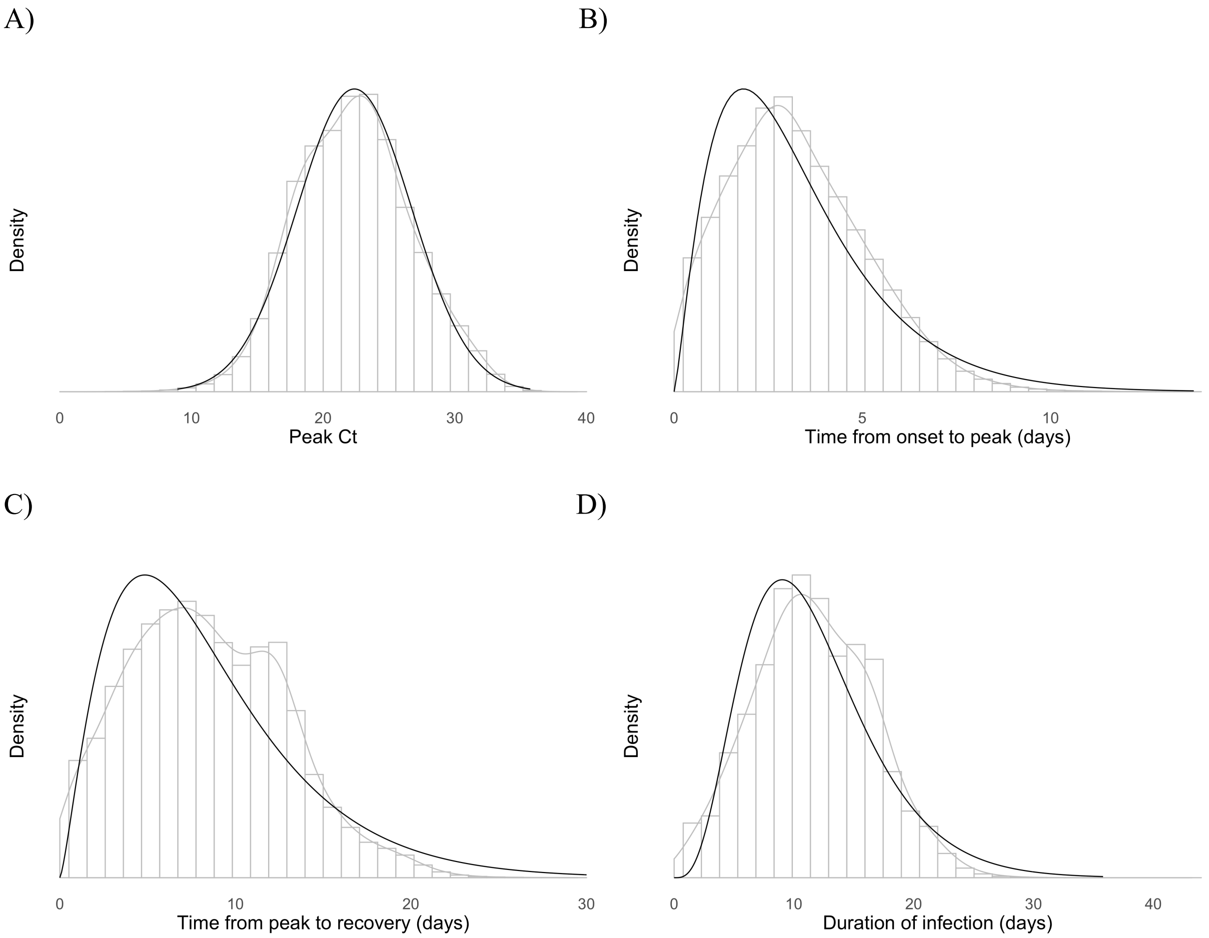

S13 Fig. Individual-level peak Ct value and waiting time distributions. Histograms (grey bars) of 10,000 posterior draws from the distributions for peak Ct value (A), time from onset to peak (B), time from peak to recovery (C), and total duration of infection (D) across the 46 individuals with an acute infection. Grey curves are kernel density estimators to more clearly exhibit the shape of the histogram. Black curves represent the best-fit normal (A) or gamma (B, C, D) distributions to the histograms. The duration of infection is the sum of the time from onset to peak and the time from peak to recovery. The best-fit normal distribution to the posterior peak Ct value distribution had mean 22.3 and standard deviation 4.2; the best-fit gamma distribution to the proliferation stage duration had shape parameter 2.3 and inverse scale parameter 0.7; the best-fit gamma distribution to the clearance stage duration had shape parameter 2.4 and inverse scale parameter 0.3; and the best-fit gamma distribution to the total duration of infection had shape parameter 4.3 and inverse scale parameter 0.4. Alternatively, the proliferation, clearance, and duration of infection distributions can be summarized as log-normal distributions. The best-fit log-normal distribution to the proliferation stage duration had location parameter $\mu = 0.93$ and scale parameter $\sigma = 0.82$; the best-fit log-normal distribution to the clearance stage duration had location parameter $\mu = 1.9$ and scale parameter $\sigma = 0.83$; and the best-fit log-normal distribution to the total duration of infection had location parameter $\mu = 2.3$ and scale parameter $\sigma = 0.53$. Underlying data are available at https://github.com/gradlab/CtTrajectories/tree/main/output/params_df_split.csv[10]
