# Supplementary material for: Viral dynamics of acute SARS-CoV-2 infection and applications to diagnostic and public health strategies
Source: PLoS Biol. 2021 Jul 12;19(7):e3001333. doi: 10.1371/journal.pbio.3001333 (PMC8297933; doi:10.1371/journal.pbio.3001333)
Supplement: S14 Fig — Posterior peak viral concentration distribution for symptomatic (red) and asymptomatic (blue) individuals (A) and for all individuals combined (B). Underlying data are available at https://github.com/gradlab/CtTrajectories/tree/main/output/params_df_split.csv (A) and https://github.com/gradlab/CtTrajectories/tree/main/output/params_df_combined.csv (B). (PDF) [file pbio.3001333.s014.pdf]

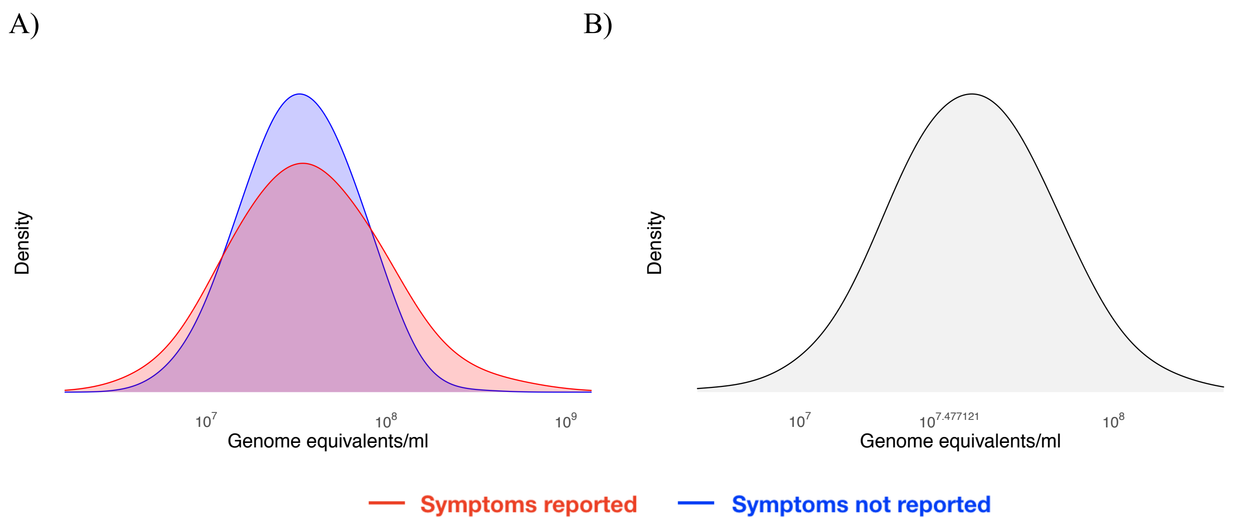

S14 Fig. Peak viral concentration and overall posterior viral concentration trajectories in terms of genome equivalents per ml. Posterior peak viral concentration distribution for symptomatic (red) and asymptomatic (blue) individuals (A) and for all individuals combined (B). Undelrying data are available at
https://github.com/gradlab/CtTrajectories/tree/main/output/params_df_split.csv[10] (A) and
https://github.com/gradlab/CtTrajectories/tree/main/output/params_df_combined.csv[10] (B)
