# Supplementary material for: Viral dynamics of acute SARS-CoV-2 infection and applications to diagnostic and public health strategies
Source: PLoS Biol. 2021 Jul 12;19(7):e3001333. doi: 10.1371/journal.pbio.3001333 (PMC8297933; doi:10.1371/journal.pbio.3001333)
Supplement: S15 Fig — Points depict the Ct values for SARS-CoV-2 nasal swab samples that were tested in both the Florida and Yale labs. Ct values from Florida represent Target 1 (ORF1ab) on the Roche cobas system, and Ct values from Yale represent N1 in the Yale multiplex assay. The solid black line depicts the best-fit linear regression (intercept = −6.25, slope = 1.34, R2 = 0.86). The dashed black line marks the 1–1 line where the points would be expected to fall if the 2 labs produced identical results. Underlying data are available at https://github.com/gradlab/CtTrajectories/tree/main/figure_data/FigS15. (PDF) [file pbio.3001333.s015.pdf]

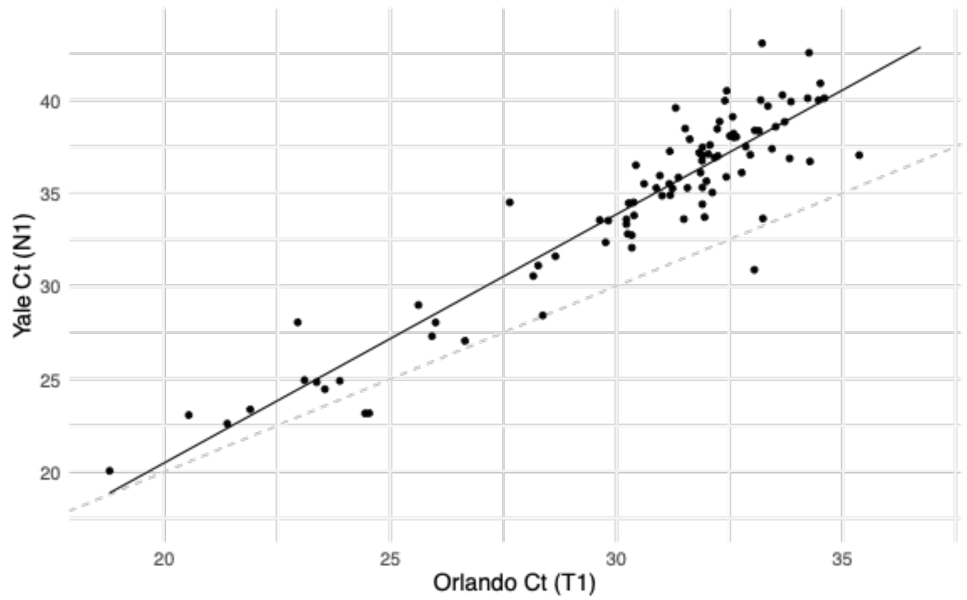

S15 Fig. Ct values from the Yale and Florida labs. Points depict the Ct values for SARS-CoV-2 nasal swab samples that were tested in both Florida and Yale labs. Ct values from Florida represent Target 1 (ORF1ab) on the Roche cobas system, and Ct values from Yale represent N1 in the Yale multiplex assay. The solid black line depicts the best-fit linear regression (intercept = –6.25, slope = 1.34, R^2 = 0.86). The dashed black line marks the 1-1 line where the points would be expected to fall if the two labs were identical. Underlying data are available at https://github.com/gradlab/CtTrajectories/tree/main/figure_data/FigS15[10]
