# Supplementary material for: Viral dynamics of acute SARS-CoV-2 infection and applications to diagnostic and public health strategies
Source: PLoS Biol. 2021 Jul 12;19(7):e3001333. doi: 10.1371/journal.pbio.3001333 (PMC8297933; doi:10.1371/journal.pbio.3001333)
Supplement: S16 Fig — Points depict the residual after removing the best-fit linear trend in the relationship between the Yale and Florida Ct values. Underlying data are available at https://github.com/gradlab/CtTrajectories/tree/main/figure_data/FigS16. (PDF) [file pbio.3001333.s016.pdf]

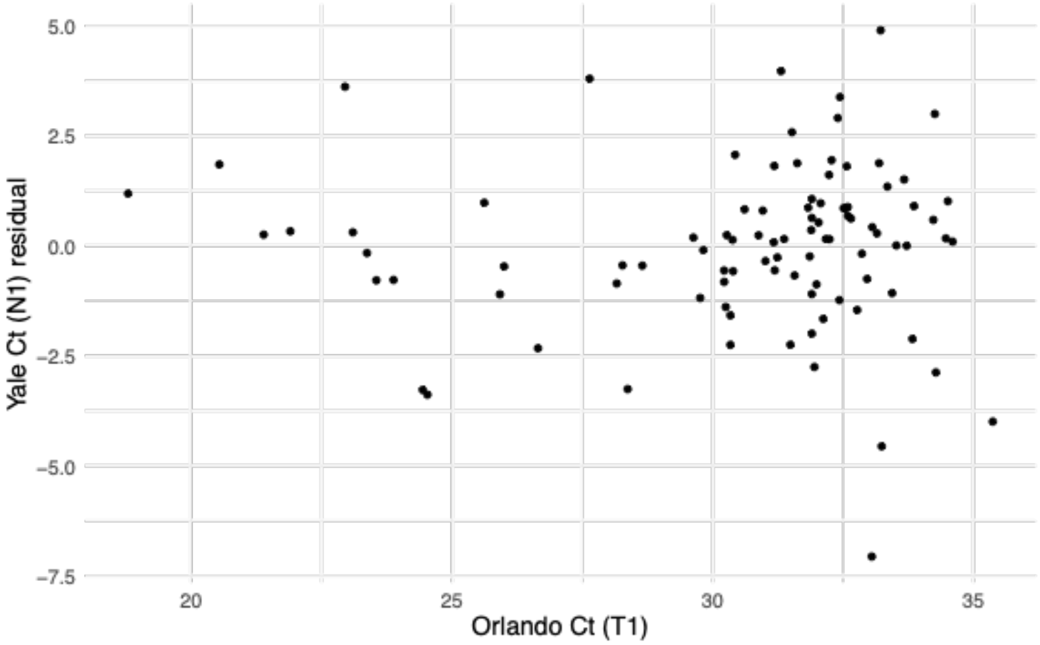

S16 Fig. Residuals from the Yale/Florida Ct regression. Points depict the residual after removing the best-fit linear trend in the relationship between the Yale and Florida Ct values. Underlying data are available at

https://github.com/gradlab/CtTrajectories/tree/main/figure_data/FigS16[10]
