# Supplementary material for: Viral dynamics of acute SARS-CoV-2 infection and applications to diagnostic and public health strategies
Source: PLoS Biol. 2021 Jul 12;19(7):e3001333. doi: 10.1371/journal.pbio.3001333 (PMC8297933; doi:10.1371/journal.pbio.3001333)
Supplement: S17 Fig — The residuals were standardized (by subtracting the mean of all residuals from each residual and then dividing each residual by the standard deviation of all residuals) before being compared with the theoretical quantiles of a normal distribution with mean 0 and standard deviation 1. The points depict the empirical quantiles of the data points, and the line depicts where the points would be expected to fall if they were drawn from a standard normal distribution. Underlying data are available at https://github.com/gradlab/CtTrajectories/tree/main/figure_data/FigS17. (PDF) [file pbio.3001333.s017.pdf]

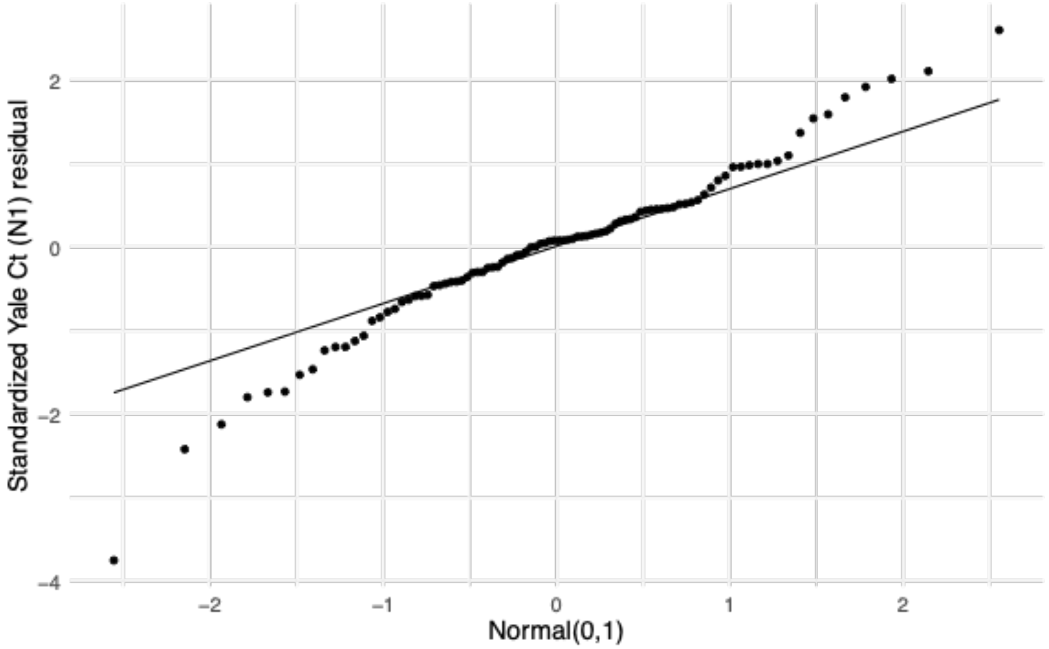

S17 Fig. QQ plot of the residuals from the Yale/Florida Ct regression. The residuals were standardized (subtracted the mean and divided by the standard deviation) before comparing with the theoretical quantiles of a normal distribution with mean 0 and standard deviation 1. The points depict the empirical quantiles of the data points and the line depicts the where the points would be expected to fall if they were drawn from a standard normal distribution. Underlying data are available at

https://github.com/gradlab/CtTrajectories/tree/main/figure_data/FigS17[10]
