# Supplementary material for: Viral dynamics of acute SARS-CoV-2 infection and applications to diagnostic and public health strategies
Source: PLoS Biol. 2021 Jul 12;19(7):e3001333. doi: 10.1371/journal.pbio.3001333 (PMC8297933; doi:10.1371/journal.pbio.3001333)
Supplement: S18 Fig — (A and C) Effective sensitivity and (B and D) number of infectious individuals expected to attend a gathering of size 1,000 assuming a population prevalence of 2% infectious individuals and a test with limit of detection of 40 Ct and 1% sampling error probability (red) and a test with limit of detection of 35 Ct and 5% sampling error probability (blue) administered between 0 and 3 days before the gathering. For (A) and (B) individuals are assumed to be infectious when their Ct value is below 35. For (C) and (D) individuals are assumed to be infectious when their Ct value is below 20. Shaded bands represent 90% prediction intervals generated from the quantiles of 1,000 simulated events and capture uncertainty both in the number of infectious individuals who would arrive at the event in the absence of testing and in the probability that the test successfully identifies infectious individuals. The dashed lines in (B) and (D) depict the expected number of infectious individuals who would attend the gathering in the absence of testing. Setting the infectiousness threshold at higher viral concentration (20 Ct versus 35 Ct) makes it less likely that an individual will become infectious at all during the course of their acute infection, leading to the lower expected number of infectious individuals at the gathering in (D) versus (B). Underlying data are available at https://github.com/gradlab/CtTrajectories/tree/main/figure_data/FigS18. (PDF) [file pbio.3001333.s018.pdf]

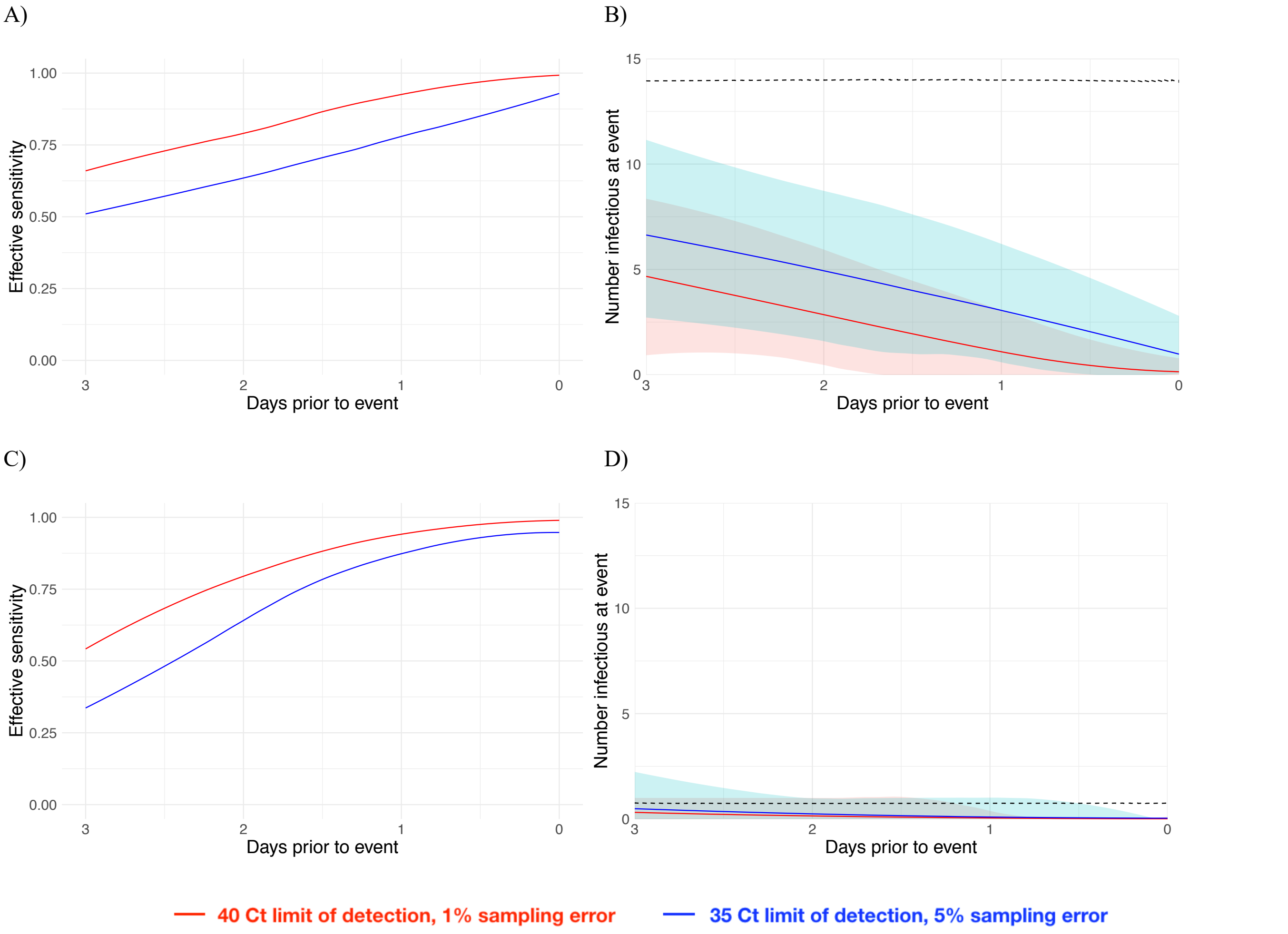

S18 Fig. Effective sensitivity and expected number of infectious gathering attendees given a pre-gathering test and varying infectiousness thresholds. (A, C) Effective sensitivity and (B, D) number of individuals expected to attend a gathering of size 1,000 assuming a population prevalence of 2% infectious individuals and a test with limit of detection of 40 Ct and 1% sampling error probability (red) and a test with limit of detection of 35 Ct and 5% sampling error probability (blue) administered between 0 and 3 days before the gathering. For (A) and (B) an individual is assumed to be infectious when their Ct value is below 35. For (C) and (D) an individual is assumed to be infectious when their Ct value is below 20. Shaded bands represent 90% prediction intervals generated from the quantiles of 1,000 simulated events and capture uncertainty both in the number of infectious individuals who would arrive at the event in the absence of testing and in the probability that the test successfully identifies infectious individuals. The dashed lines in (B) and (D) depict the expected number of infectious individuals who would attend the gathering in the absence of testing. An infectiousness threshold at higher viral concentration (20 Ct *vs.* 35 Ct) makes it less likely that an individual will become infectious at all during the course of their acute infection, leading to the relatively lower expected number of infectious individuals at the gathering in panel (D) *vs.* panel (B). Underlying data are available at https://github.com/gradlab/CtTrajectories/tree/main/figure_data/FigS18[10]
