# Supplementary material for: Viral dynamics of acute SARS-CoV-2 infection and applications to diagnostic and public health strategies
Source: PLoS Biol. 2021 Jul 12;19(7):e3001333. doi: 10.1371/journal.pbio.3001333 (PMC8297933; doi:10.1371/journal.pbio.3001333)
Supplement: S19 Fig — For a given viral trajectory conditioned on infectiousness during a gathering, there is a wider range of possible proliferation onset times when the infectiousness threshold is low (blue) versus when the infectiousness threshold is high (red). Additionally, the range of possible onset times for the low infectiousness threshold versus the high infectiousness threshold is skewed to the left since the clearance time is longer than the proliferation time. Because of this, a low infectiousness threshold makes it easier for a pre-gathering test to pick up a trajectory that would be infectious at the time of the gathering. Conversely, a high infectiousness threshold shortens the window of possible onset times that guarantee infectiousness during the gathering, making it more difficult for a pre-gathering test to detect the trajectory. This is reflected in the steeper decline in the effective sensitivity for a high infectiousness threshold (Ct = 20) than for a low infectiousness threshold (Ct = 35) (see S18A and S18C Fig). (PDF) [file pbio.3001333.s019.pdf]

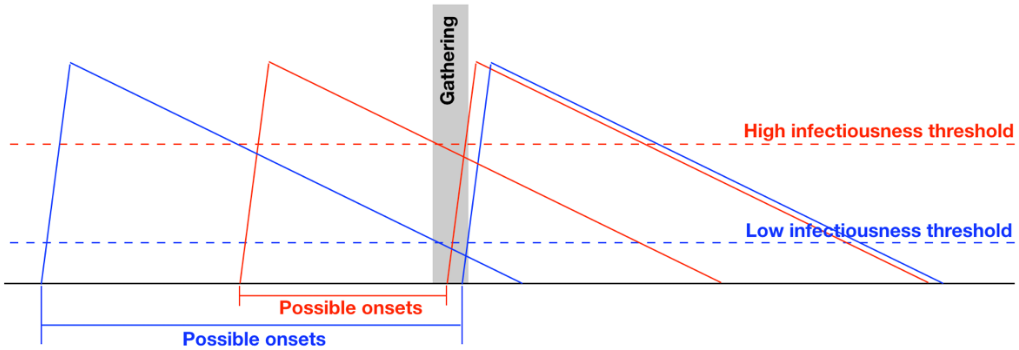

S19 Fig. Illustration of why effective sensitivity declines more sharply with testing delays for high vs. low infectiousness thresholds. For a given viral trajectory conditioned on infectiousness during a gathering, there is a wider range of possible proliferation onset times when the infectiousness threshold is low (blue) *vs.* when the infectiousness threshold is high (red). Additionally, the range of possible onset times for the low infectiousness threshold *vs.* the high infectiousness threshold is skewed to the left since the clearance time is longer than the proliferation time. Because of this, a low infectiousness threshold makes it easier for a pre-gathering test to pick up a trajectory that would be infectious at the time of the gathering. Conversely, a high infectiousness threshold shortens the window of possible onset times that guarantee infectiousness during the gathering, making it more difficult for a pre-gathering test to detect the trajectory. This is reflected in the steeper decline in the effective sensitivity for a high infectiousness threshold (Ct = 20) than for a low infectiousness threshold (Ct = 35) (**S18 Fig, A/C**).
