# Supplementary material for: Viral dynamics of acute SARS-CoV-2 infection and applications to diagnostic and public health strategies
Source: PLoS Biol. 2021 Jul 12;19(7):e3001333. doi: 10.1371/journal.pbio.3001333 (PMC8297933; doi:10.1371/journal.pbio.3001333)
Supplement: S1 Table — Synthetic T7 RNA transcripts corresponding to a 1,363-base-pair segment of the SARS-CoV-2 nucleocapsid gene were serially diluted from 106 to 100 and evaluated in duplicate with RT-qPCR. The best-fit linear regression of the average Ct on the log10-transformed standard values had slope −3.60971 and intercept 40.93733 (R2 = 0.99). (PDF) [file pbio.3001333.s020.pdf]

S1 Table. Standard curve relationship between genome equivalents and Ct values. Synthetic T7 RNA transcripts corresponding to a 1,363 base pair segment of the SARS-CoV-2 nucleocapsid gene were serially diluted from 10^6-10^0 and evaluated in duplicate with RT-qPCR. The best-fit linear regression of the average Ct on the log10-transformed standard values had slope -3.60971 and intercept 40.93733 (R^2 = 0.99).

Standard (GE/ul)	Replicate 1 (Ct)	Replicate 2 (Ct)	Average Ct
**10^6**	19.3	19.7	19.5
**10^5**	23.0	21.2	22.1
**10^4**	26.9	26.7	26.8
**10^3**	30.6	30.4	30.5
**10^2**	34.0	34.0	34.0
**10^1**	37.2	36.6	36.9
**10^0**	N/A	39.9	39.9
