# Supplementary material for: Viral dynamics of acute SARS-CoV-2 infection and applications to diagnostic and public health strategies
Source: PLoS Biol. 2021 Jul 12;19(7):e3001333. doi: 10.1371/journal.pbio.3001333 (PMC8297933; doi:10.1371/journal.pbio.3001333)
Supplement: S2 Table — (PDF) [file pbio.3001333.s021.pdf]

S2 Table. Viral dynamic parameters for sensitivity analysis 1, omitting person 3047

**Parameter**	**Mean, symptoms [95% CI]**	**Mean, no symptoms [95% CI]**
Peak Ct	22.3 [19.4, 25.1]	22.2 [20, 24.3]
Peak viral concentration (RNA copies/ml/day)	7.6 [6.8, 8.4]	7.6 [7, 8.2]
Proliferation duration (days)	3.3 [2, 4.9]	3.6 [2.7, 4.7]
Proliferation rate (Ct/day)	5.7 [3.5, 9.2]	5.1 [3.7, 6.8]
Proliferation rate (RNA copies/ml/day)	1.6 [1, 2.6]	1.4 [1, 1.9]
Clearance duration (days)	11.1 [8.2, 14.1]	8.0 [6.3, 9.9]
Clearance rate (Ct/day)	1.6 [1.2, 2.3]	2.2 [1.7, 2.9]
Clearance rate (RNA copies/ml/day)	0.5 [0.3, 0.6]	0.6 [0.5, 0.8]
Infection duration (days)	14.3 [11.3, 17.5]	11.6 [9.7, 13.6]
