# Supplementary material for: Viral dynamics of acute SARS-CoV-2 infection and applications to diagnostic and public health strategies
Source: PLoS Biol. 2021 Jul 12;19(7):e3001333. doi: 10.1371/journal.pbio.3001333 (PMC8297933; doi:10.1371/journal.pbio.3001333)
Supplement: S3 Table — (PDF) [file pbio.3001333.s022.pdf]

S3 Table. Viral dynamic parameters for sensitivity analysis 2, assuming 95% PCR sensitivity or 5% probability of false negative.

Parameter	Mean, symptoms [95% CI]	Mean, no symptoms [95% CI]
Peak Ct	22.2 [19.1, 25.1]	22.4 [20.1, 24.7]
Peak viral concentration (RNA copies/ml/day)	7.6 [6.8, 8.4]	7.6 [7, 8.2]
Proliferation duration (days)	3.8 [2, 5.9]	3.7 [2.7, 4.8]
Proliferation rate (Ct/day)	5.1 [2.9, 9.2]	4.9 [3.5, 6.6]
Proliferation rate (RNA copies/ml/day)	1.6 [1, 2.6]	1.4 [1, 1.9]
Clearance duration (days)	10.7 [7.7, 13.9]	7.9 [6.1, 9.8]
Clearance rate (Ct/day)	1.7 [1.2, 2.4]	2.3 [1.7, 3]
Clearance rate (RNA copies/ml/day)	0.5 [0.3, 0.6]	0.6 [0.5, 0.8]
Infection duration (days)	14.5 [11.2, 17.8]	11.6 [9.6, 13.7]
