# Supplementary material for: Viral dynamics of acute SARS-CoV-2 infection and applications to diagnostic and public health strategies
Source: PLoS Biol. 2021 Jul 12;19(7):e3001333. doi: 10.1371/journal.pbio.3001333 (PMC8297933; doi:10.1371/journal.pbio.3001333)
Supplement: S4 Table — (PDF) [file pbio.3001333.s023.pdf]

S4 Table. Viral dynamic parameters for sensitivity analysis 3, removing upper bounds for proliferation and clearance times.

**Parameter**	**Mean, symptoms [95% CI]**	**Mean, no symptoms [95% CI]**
Peak Ct	22.3 [19.1, 25.1]	22.4 [20.2, 24.6]
Peak viral concentration (RNA copies/ml/day)	7.6 [6.8, 8.4]	7.5 [6.9, 8.1]
Proliferation duration (days)	3.4 [2.1, 5.2]	3.5 [2.5, 4.5]
Proliferation rate (Ct/day)	5.4 [3.3, 8.8]	5.2 [3.7, 7.1]
Proliferation rate (RNA copies/ml/day)	1.5 [0.9, 2.4]	1.4 [1, 2]
Clearance duration (days)	10.8 [7.7, 14.1]	7.8 [6.1, 9.8]
Clearance rate (Ct/day)	1.7 [1.2, 2.4]	2.3 [1.7, 3.0]
Clearance rate (RNA copies/ml/day)	0.5 [0.3, 0.7]	0.6 [0.5, 0.8]
Infection duration (days)	14.3 [11.1, 17.7]	11.3 [9.3, 13.4]
