# Supplementary material for: Viral dynamics of acute SARS-CoV-2 infection and applications to diagnostic and public health strategies
Source: PLoS Biol. 2021 Jul 12;19(7):e3001333. doi: 10.1371/journal.pbio.3001333 (PMC8297933; doi:10.1371/journal.pbio.3001333)
Supplement: S5 Table — (PDF) [file pbio.3001333.s024.pdf]

S5 Table. Viral dynamic parameters for sensitivity analysis 4, using "low" priors for the proliferation and clearance times (mean 3.5 and 7.5 days, respectively).

**Parameter**	**Mean, symptoms [95% CI]**	**Mean, no symptoms [95% CI]**
Peak Ct	22.2 [19, 25.1]	22.4 [20.2, 24.6]
Peak viral concentration (RNA copies/ml/day)	7.6 [6.8, 8.5]	7.5 [6.9, 8.1]
Proliferation duration (days)	3.1 [1.9, 4.6]	3.2 [2.3, 4.2]
Proliferation rate (Ct/day)	6.1 [3.7, 9.8]	5.6 [4, 7.6]
Proliferation rate (RNA copies/ml/day)	1.7 [1, 2.7]	1.5 [1.1, 2.1]
Clearance duration (days)	10.0 [6.9, 13.2]	7.5 [5.8, 9.4]
Clearance rate (Ct/day)	1.8 [1.3, 2.7]	2.4 [1.8, 3.1]
Clearance rate (RNA copies/ml/day)	0.5 [0.4, 0.8]	0.7 [0.5, 0.9]
Infection duration (days)	13.1 [9.9, 16.4]	10.8 [8.9, 12.8]
