# Supplementary material for: Viral dynamics of acute SARS-CoV-2 infection and applications to diagnostic and public health strategies
Source: PLoS Biol. 2021 Jul 12;19(7):e3001333. doi: 10.1371/journal.pbio.3001333 (PMC8297933; doi:10.1371/journal.pbio.3001333)
Supplement: S6 Table — (PDF) [file pbio.3001333.s025.pdf]

S6 Table. Viral dynamic parameters for sensitivity analysis 5, using "high" priors for the proliferation and clearance times (mean 10.5 days and 22.5 days, respectively)

Parameter	Mean, symptoms [95% CI]	Mean, no symptoms [95% CI]
Peak Ct	22.3 [19.2, 25.2]	22.3 [20.2, 24.4]
Peak viral concentration (RNA copies/ml/day)	7.6 [6.8, 8.4]	7.5 [7, 8.1]
Proliferation duration (days)	3.7 [2.2, 5.4]	3.6 [2.7, 4.7]
Proliferation rate (Ct/day)	5.1 [3.1, 8.3]	5.0 [3.6, 6.8]
Proliferation rate (RNA copies/ml/day)	1.4 [0.9, 2.3]	1.4 [1, 1.9]
Clearance duration (days)	11.6 [8.5, 14.7]	8.0 [6.3, 10]
Clearance rate (Ct/day)	1.6 [1.1, 2.2]	2.2 [1.7, 2.9]
Clearance rate (RNA copies/ml/day)	0.4 [0.3, 0.6]	0.6 [0.5, 0.8]
Infection duration (days)	15.2 [12, 18.7]	11.6 [9.7, 13.7]
