# Supplementary material for: Viral dynamics of acute SARS-CoV-2 infection and applications to diagnostic and public health strategies
Source: PLoS Biol. 2021 Jul 12;19(7):e3001333. doi: 10.1371/journal.pbio.3001333 (PMC8297933; doi:10.1371/journal.pbio.3001333)
Supplement: S1 Text — (PDF) [file pbio.3001333.s026.pdf]

S1 Text.

Supplemental Methods.

Additional testing protocol details.

Clinical samples were obtained by combined swabs of the anterior nares and oropharynx administered by a trained provider. The samples were initially tested by either Quest Diagnostics (while teams were in local markets using the Quest SARS-CoV-2 RT-qPCR[1]) or BioReference Laboratories (while teams were in Orlando using the cobas SARS-CoV-2 test[2]). Viral transport media from positive samples were sent to Yale University for subsequent RT-qPCR testing using a multiplexed version of the assay from the US Centers for Disease Control and Prevention[3] to normalize Ct values across testing platforms. To account for the different calibration of the testing instruments, we used a linear conversion (**S15 Fig – S17 Fig**, **Methods: Converting Ct values**) to adjust any samples not processed at Yale University to the Yale laboratory scale. Subsequent analysis is based on the N1 Ct value from the Yale multiplex assay and on the adjusted Roche cobas target 1 assay.

Residual viral transport media (VTM) from Quest Diagnostics or BioReference Laboratories were shipped overnight to Yale on dry ice. VTM was thawed on ice and 300 µL was used for RNA extraction using the MagMAX Viral/Pathogen Nucleic Acid Isolation Kit and the KingFisher Flex robot (Thermo Fisher Scientific, Waltham, MA[4]). Total nucleic acid was eluted into 75ul of elution buffer and SARS-CoV-2 RNA was quantified from 5 µL of extracted total RNA using a multiplexed version of the CDC RT-qPCR assay that contains the 2019-nCoV_N1 (N1), 2019-nCoV-N2 (N2), and human RNase P (RP) primer-probe sets[3]. The RT-qPCR was performed using the Luna Universal Probe One-Step RT-qPCR Kit (New England Biolabs, Ipswich, MA, US) and the following thermocycler conditions: (1) reverse transcription for 10 minutes at 55°C, (2) initial denaturation for 1 min at 95°C, and PCR for 45 cycles of 10 seconds at 95°C and 30 seconds at 55°C on the CFX96 qPCR machine (Bio-Rad, Hercules, CA, US).

Converting Ct values. Most (n = 226) of the 312 positive samples in the raw dataset underwent RT-qPCR at the Yale laboratory. We used the Yale Ct value whenever it was available. Still, 86 samples underwent initial diagnostic testing at BioReference Laboratories but not confirmatory testing at the Yale laboratory. Both platforms rely on a multiplex RT-qPCR strategy. The two testing platforms yield slightly different Ct values, as evidenced by the 94 samples the underwent RT-qPCR at both facilities (**S15 Fig**). For comparison between platforms, target 1 from the Roche cobas assay, which is specific to SARS-CoV-2, and the N1 target from the Yale multiplex assay were used. For the 86 samples that were not processed at the Yale laboratory, we adjusted the Ct values using the best-fit (minimum sum of squares) linear regression between the initial Ct value and the Yale Ct value for the samples that were processed in both facilities. To do so, we estimated the coefficients β_0 and β_1 in the following regression equation:

$$y_i = \beta_0 + \beta_1 x_i + \epsilon_i$$

Here, y_i denotes the ith Ct value from Yale, x_i denotes the ith Ct value from the initial test, and ε_i is an error term with mean 0 and constant variance across all samples. The resulting fit (**S15 Fig**) was strong (R^2 = 0.86) with homoscedastic residuals (**S16 Fig**) that are approximately normally distributed, as evidenced by a Q-Q plot (**S17 Fig**).

Data parsing. The raw dataset included 3,207 test results for 102 individuals. We excluded 21 individuals who had 5 or fewer tests, since the data for these individuals were too sparse to reliably infer a Ct trajectory. We also excluded 13 individuals who did not record any Ct values that surpassed the RT-qPCR limit of detection (40). We removed 146 entries for which the test result was recorded as 'positive' but there was no associated Ct value; these tests were initially conducted on an instrument that provided only a binary diagnosis and the samples were not available for confirmatory testing. This left 2,411 total tests for 68 individuals for the main analysis. The median number of tests administered to each of the 68 individuals was 41 (IQR [14, 51]; Range [5, 70]). The median number of Ct values with viral concentration above the limit of detection recorded for each person was 3 (IQR [2, 4]; Range [1, 9]). We trivially shifted the date indices so that date 0 corresponded to the time of the minimum Ct. We set the Ct value for

negative tests equal to the limit of detection. For the statistical analysis, we removed any sequences of 3 or more consecutive negative tests to avoid overfitting to these trivial values. Acute infections were defined as probable novel infections that reached a minimum Ct value of below 35.

Model fitting.

We assumed that the viral concentration trajectories consisted of a proliferation phase, with exponential growth in viral RNA concentration, followed by a clearance phase characterized by exponential decay in viral RNA concentration[5]. Since Ct values are roughly proportional to the negative logarithm of viral concentration[6], this corresponds to a linear decrease in Ct followed by a linear increase. We therefore constructed a piecewise-linear regression model to estimate the peak Ct value, the time from infection onset to peak (*i.e.* the duration of the proliferation stage), and the time from peak to infection resolution (*i.e.* the duration of the clearance stage). This idealized trajectory is depicted in **S6 Fig.** The trajectory may be represented by the equation

$$E[Ct(t)] = \begin{cases} \text{l.o.d} & t \leq t_o \\ \text{l.o.d} - \frac{\chi}{t_p - t_o}(t - t_o) & t_o < t \leq t_p \\ \text{l.o.d} - \chi + \frac{\chi}{t_r - t_p}(t - t_p) & t_p < t \leq t_r \\ \text{l.o.d} & t > t_r \end{cases}$$

Here, E[$Ct(t)$] represents the expected value of the Ct at time t, "l.o.d" represents the RT-qPCR limit of detection, χ is the absolute difference in Ct between the limit of detection and the peak (lowest) Ct, and t_o, t_p, and t_r are the onset, peak, and recovery times, respectively.

Before fitting, we re-parametrized the model using the following definitions:

- $\Delta Ct(t)$ = l.o.d. – $Ct(t)$ is the difference between the limit of detection and the observed Ct value at time t.
- $\omega_p = t_p - t_o$ is the duration of the proliferation stage (first potential detectability to peak viral concentration).
- $\omega_r = t_r - t_p$ is the duration of the clearance stage (peak viral concentration to resolution of acute infection).

We constrained $0 \leq \omega_p \leq 14$ days and $0 \leq \omega_r \leq 30$ days to prevent inferring unrealistically large values for these parameters for trajectories that were missing data prior to the peak and after the peak, respectively. We also constrained $0 \leq \chi \leq 40$ as Ct values can only take values between 0 and the limit of detection (40).

We next assumed that the observed $\Delta Ct(t)$ could be described the following mixture model:

$$\Delta Ct(t) \sim \lambda \text{ Normal}(E[\Delta Ct(t)], \sigma(t)) + (1-\lambda) \text{ Exponential}(\log(10))\Big]_0^{\text{l.o.d}}$$

where E[$\Delta Ct(t)$] = l.o.d. - E[$Ct(t)$] and λ is the sensitivity of the q-PCR test, which we fixed at 0.99. The bracket term on the right-hand side of the equation denotes that the distribution was truncated to ensure Ct values between 0 and the limit of detection. This model captures the scenario where most observed Ct values are normally distributed around the expected trajectory with standard deviation $\sigma(t)$, yet there is a small (1%) probability of an exponentially-distributed false negative near the limit of detection. The log(10) rate of the exponential distribution was chosen so that 90% of the mass of the distribution sat below 1 Ct unit and 99% of the distribution sat below 2 Ct units, ensuring that the distribution captures values distributed at or near the limit of detection. We did not estimate values for λ or the exponential rate because they were not of interest in this study; we simply needed to include them to account for some small probability mass that persisted near the limit of detection to allow for the possibility of false negatives.

For the 86 samples that were not tested in the Yale laboratory, we included additional uncertainty in the observed Ct value by inflating $\sigma(t)$, such that

$$\sigma(t) = (\tilde{\sigma}^2 + \epsilon^2 I_{adj})^{1/2}$$

Here, σ(tilde) is a constant, ε is the standard deviation of the residuals from the linear fit between the initial test and the Yale laboratory test, and I_{adj} is an indicator variable that is 1 if the sample at time t was adjusted and 0 otherwise.

We used a hierarchical structure to describe the distributions of ω_p, ω_r, and χ for each individual based on their respective population means $\mu_{\omega p}$, $\mu_{\omega r}$, and μ_χ and population standard deviations $\sigma_{\omega p}$, $\sigma_{\omega r}$, and σ_χ such that

$\omega_p \sim \text{Normal}(\mu_{\omega p}, \sigma_{\omega p})$

$\omega_r \sim \text{Normal}(\mu_{\omega r}, \sigma_{\omega r})$

$\chi \sim \text{Normal}(\mu_\chi, \sigma_\chi)$

We inferred separate population means ($\mu_\bullet$) for symptomatic and asymptomatic individuals. We used a Hamiltonian Monte Carlo fitting procedure implemented in Stan (version 2.24) [7] and R (version 3.6.2)[8] to estimate the individual-level parameters ω_p, ω_r, χ, and t_p as well as the population-level parameters *σ(tilde)*, $\mu_{\omega p}$, $\mu_{\omega r}$, μ_χ, $\sigma_{\omega p}$, $\sigma_{\omega r}$, and σ_χ. We used the following priors:

Hyperparameters:

σ(tilde) ~ Cauchy(0, 5) [0, ∞]

$\mu_{\omega p}$ ~ Normal(14/2, 14/6) [0, 14]

$\mu_{\omega r}$ ~ Normal(30/2, 30/6) [0, 30]

μ_χ ~ Normal(40/2, 40/6) [0, 40]

$\sigma_{\omega p}$ ~ Cauchy(0, 14/tan(π(0.95-0.5))) [0, ∞]

$\sigma_{\omega r}$ ~ Cauchy(0, 30/tan(π(0.95-0.5))) [0, ∞]

σ_χ ~ Cauchy(0, 40/tan(π(0.95-0.5))) [0, ∞]

Individual-level parameters:

$\omega_p \sim$ Normal($\mu_{\omega p}$, $\sigma_{\omega p}$) [0,14]

$\omega_r \sim$ Normal($\mu_{\omega r}$, $\sigma_{\omega r}$) [0,30]

$\chi \sim$ Normal(μ_χ, σ_χ) [0,40]

$t_p \sim$ Normal(0, 2)

The values in square brackets denote truncation bounds for the distributions. We chose a vague half-Cauchy prior with scale 5 for the observation variance *σ(tilde)*. The priors for the population mean values ($\mu_\bullet$) are normally-distributed priors spanning the range of allowable values for that parameter; this prior is vague but expresses a mild preference for values near the center of the allowable range. The priors for the population standard deviations ($\sigma_\bullet$) are half Cauchy-distributed with scale chosen so that 90% of the distribution sits below the maximum value for that parameter; this prior is vague but expresses a mild preference for standard deviations close to 0.

We ran four MCMC chains for 1,000 iterations each with a target average proposal acceptance probability of 0.99. The first half of each chain was discarded as the warm-up. The Gelman R-hat statistic was less than 1.1 for all parameters except for the t_p and ω_r associated with person ID 1370, as the posterior distributions for those parameters were multi-modal (see **S11 Fig – S12 Fig**). This indicates good overall mixing of the chains. There were fewer than 10 divergent iterations (<0.1% of the transitions after warm-up), indicating good exploration of the parameter space. The posterior distributions for μ_χ, $\mu_{\omega p}$, and $\mu_{\omega r}$, estimated separately for symptomatic and asymptomatic individuals, are reported in **Fig 3** (main text). We fit a second model that did not distinguish between symptomatic and asymptomatic individuals. The posterior distributions for these same parameters under this model are depicted in **S8 Fig**. The posterior distributions for the individual-level parameters ω_p, ω_r, and χ are depicted in **S9 Fig – S11 Fig,** with 500 sampled trajectories from these posterior distributions for each individual depicted in **S12 Fig.** The overall combined posterior distributions for the individual-level parameters ω_p, ω_r, and χ are depicted in **S13 Fig.**

We estimated the best-fit normal (for χ) and gamma (for ω_p and ω_r) distributions using the 'fitdistrplus' package implemented in R (version 3.6.2)[8].

Sensitivity analyses.

To check the model's robustness, we performed a variety of sensitivity analyses. First, we omitted the viral trajectory from individual 3047 since their trajectory consisted of a long series of high-Ct positive tests which might be better characterized as a persistent infection rather than an acute infection. Second, we reduced the assumed PCR sensitivity (1–false negative rate) from 0.99 to 0.95. Third, we removed the upper-bound constraints on the proliferation and clearance durations. Fourth, we re-fit the model using "low" prior distributions for the proliferation and clearance distributions that had mean 14/4=3.5 days and 30/4=7.5 days respectively (same prior standard deviations). Fifth, we re-fit the model using "high" prior distributions for the proliferation and clearance distribution that had mean 14*3/4=10.5 days and 30*3/4=22.5 days respectively (same prior standard deviation). The posterior means and 95% credible intervals are listed in **S2 Table – S6 Table.** The posterior means and credible intervals are consistent throughout. For the final two scenarios, the "low" and "high" priors shift the posterior means for the proliferation and clearance times down and up, respectively, especially for the symptomatic group which had fewer samples. Still, the overall findings of a consistent proliferation stage length and a somewhat longer clearance stage remain consistent.

Converting Ct values to viral genome equivalents.

CT values were fitted to a standard curve in order to convert Ct value data to RNA copies or genome equivalents (GE). Synthetic T7 RNA transcripts corresponding to a 1,363 b.p. segment of the SARS-CoV-2 nucleocapsid gene were serially diluted from 10^6-10^0 GE/µl in duplicate to generate a standard curve[9] **(S1 Table)**. The average Ct value for each dilution was used to calculate the slope (-3.60971) and intercept (40.93733) of the linear regression of Ct on log-10 transformed standard RNA concentration, and Ct values from subsequent RT-qPCR runs were converted to GE using the following equation:

$$\log_{10}([\text{RNA}]) = (Ct - 40.93733)/(-3.60971) + \log_{10}(250)$$

Here, [RNA] represents the RNA concentration in GE/ml. The $\log_{10}(250)$ term accounts for the extraction (300 μl) and elution (75 μl) volumes associated with processing the clinical samples as well as the 1,000 μl/ml unit conversion.

Inferring the stage of infection using single and paired Ct values

To determine whether individual or paired Ct values can reveal a patient's stage of infection, we measured the frequency with which a Ct value falling within a 5-unit band, possibly followed by a second Ct value of higher or lower magnitude, was associated with the proliferation stage, the clearance stage, or the persistent stage. First, we assigned to each positive test the probability that it was collected during each of the three stages of infection. To do so, we began with the positive samples from the 46 individuals with acute infections and calculated the frequency with which each sample sat within the proliferation stage, the clearance stage, or the persistent stage (*i.e.,* neither the proliferation nor the clearance stage) across 10,000 posterior parameter draws for that person. For the remaining 22 individuals, all positive samples were assigned to the persistent stage. Next, we calculated the probability that a Ct value falling within a 5-unit window corresponded to an active infection (*i.e.,* either the proliferation or the clearance stage) by summing the proliferation and clearance probabilities for all positive samples with that window and dividing by the total number of positive samples in the window. We considered windows with midpoints spanning from Ct = 37.5 to Ct = 15.5 (**Fig 4A**). We performed a similar calculation to determine the probability that a Ct falling within a given 5-unit window corresponded to just the proliferation phase, assuming it had already been determined that the sample fell within an active infection (**Fig 4B**). Finally, to assess the information gained by conducting a second test within two days of an initial positive, we collected all positive samples with a subsequent sample (positive or negative) that was taken within two days and repeated the above calculations, separating by whether the second test had a higher or lower Ct than the first.

Calculating effective sensitivity.

The sensitivity of a test is defined as the probability that the test correctly identifies an individual who is positive for some criterion of interest. For clinical SARS-CoV-2 tests, the criterion of interest is current infection with SARS-CoV-2. However, for the time-to-event analysis presented in the main text, the criterion of interest is infectiousness at some point in the future. The effective sensitivity of a test (with respect to future infectiousness) may differ substantially from its clinical sensitivity (with respect to current infection).

The effective sensitivity of a test intended to detect future infectiousness depends on the test's characteristics (its limit of detection and sampling error rate) as well as the viral dynamics of infected individuals. To determine the effective sensitivity of a test *n* hours before an event, we first sampled 1,000 posterior draws for the proliferation time, clearance time, and peak Ct value from the MCMC fits to the viral trajectory data. We included only draws with a peak Ct ≤ 30, as we assumed Ct = 30 to be the threshold of infectiousness (individuals with a peak Ct > 30 would never be infectious according to this threshold and therefore would never satisfy the criterion of interest, *i.e.,* infectiousness at the event). We trivially defined the event's start time to be $t = 0$ and assumed that the event lasted for 3 hours. For each of the 1,000 individuals, we identified the range of possible onset times for the proliferation stage that would ensure the person would be infectious (Ct < 30) at some point during the event. We uniform-randomly drew a proliferation onset time from this range for each individual. We then simulated a test at time $-n$. Any individuals with Ct greater than the test's limit of detection at time $-n$ went undetected. Any individuals with Ct less than the test's limit of detection at time $-n$ were detected with probability (1-sampling error). The effective sensitivity was calculated as the number of individuals successfully detected divided by the total number of individuals who could have been detected (1,000). We repeated this calculation for 1-hour increments through 3 days prior to the event. We considered two tests, one with a limit of detection at 40 Ct and a sampling error of 1% (analogous to RT-qPCR) and one with a limit of detection at 35 Ct and a sampling error of 5% (analogous to a rapid antigen test). See also the schematic diagram in **S7a Fig**.

To calculate the expected number of individuals who might attend an event while infectious given a test *n* hours before the event, we simulated 1,000 events. To do so, we again drew 1,000 individual-level viral concentration

trajectories from the fitted model and drew proliferation onset times according to a random uniform distribution from the range of possible times that would allow for the person to have detectable virus (Ct < 40) during the gathering. Note that this differs from the effective sensitivity calculation, where we required individuals to be infectious (not just detectable) at the time of the event. This way, an infectiousness threshold at an extremely high viral concentration (*e.g.*, Ct = 10) would lead to very few infectious attendees, since few viral trajectories reach such low Ct values. This aligns with intuitive notions of prevalence of infection (number of detectable infections in the population) *vs.* prevalence of infectiousness (number of individuals capable of infecting others, which is expected to be lower). We counted the number of people who would have been infectious at the gathering (a) in the absence of testing and (b) given a test administered between 0 and 3 days prior to the gathering. We considered infectiousness thresholds of Ct = 30 (main analysis), Ct = 35, and Ct = 20 (**S18 Fig).** When the infectiousness threshold is at higher viral concentrations (Ct = 20), the expected number of infectious attendees is smaller, since fewer trajectories surpass the viral concentration needed for infectiousness. The effective sensitivity also declines more quickly with testing delays when the infectiousness threshold is high (**S18 Fig**). This is attributable to the wider range of possible proliferation onset times given a low infectiousness threshold *vs*. a high infectiousness threshold (see **S19 Fig**).

The online calculator differs slightly from the above procedure. Rather than drawing directly from the posterior distributions for the MCMC fits, the calculator allows the user to input different values specifying the population distribution of proliferation times, clearance times, and peak Ct values. The proliferation times and clearance times are described by independent Gamma distributions with user-input mean and standard deviation. The peak Ct values are defined by independent normal distributions with user-input mean and standard deviation, truncated to ensure that the values lie between 0 and 40 Ct. The default values align with the best-fit values for the respective Gamma and normal distributions reported in the caption of **S13 Fig**. The effective sensitivity values and expected number of infectious attendees therefore differ slightly from the values reported in the main text due to these distributional approximations and the fact that the values are drawn independently (in the posterior draws, there is some

correlation between the parameters). Still, the calculations align closely and allow for greater flexibility in allowing the user to update the viral trajectory parameters to reflect different populations.

References

1. U.S. Food and Drug Administration. *Quest Diagnostics Infectious Disease, Inc. ("Quest Diagnostics") SARS-CoV-2 RNA Qualitative Real-Time RT-PCR Emergency Use Authorization.*; 2020.
2. U.S. Food and Drug Administration. *Roche Molecular Systems, Inc. Cobas SARS-CoV-2 Emergency Use Authorization.*; 2020.
3. Kudo E, Israelow B, Vogels CBF, Lu P, Wyllie AL, Tokuyama M, et al. Detection of SARS-CoV-2 RNA by multiplex RT-qPCR. Sugden B, ed. *PLOS Biol*. 2020;18(10):e3000867. doi:10.1371/journal.pbio.3000867
4. Ott IM, Vogels C, Grubaugh N, Wyllie AL. *Saliva Collection and RNA Extraction for SARS-CoV-2 Detection V.2.*; 2020. https://www.protocols.io/view/saliva-collection-and-rna-extraction-for-sars-cov-bh6mj9c6
5. Cleary B, Hay JA, Blumenstiel B, Gabriel S, Regev A, Mina MJ. Efficient prevalence estimation and infected sample identification with group testing for SARS-CoV-2. *medRxiv*. Published online 2020.
6. Tom MR, Mina MJ. To Interpret the SARS-CoV-2 Test, Consider the Cycle Threshold Value. *Clin Infect Dis*. 2020;02115(Xx):1-3. doi:10.1093/cid/ciaa619
7. Carpenter B, Gelman A, Hoffman MD, Lee D, Goodrich B, Betancourt M, et al. Stan : A Probabilistic Programming Language. *J Stat Softw*. 2017;76(1). doi:10.18637/jss.v076.i01
8. R Development Core Team R. R: A Language and Environment for Statistical Computing. Team RDC, ed. *R Found Stat Comput*. 2011;1(2.11.1):409. doi:10.1007/978-3-540-74686-7
9. Vogels C, Fauver J, Ott IM, Grubaugh N. *Generation of SARS-COV-2 RNA Transcript Standards for QRT-PCR Detection Assays.*; 2020. doi:10.17504/protocols.io.bdv6i69e
10. Kissler SM, Fauver JR, Mack C, Tai C, Shiue KY, Kalinich CC, et al. CtTrajectories. *GitHub*. Published online 2021. doi:10.5281/zenodo.4977246
